# Highly Effective Gene Transfection *In Vivo* by Alkylated Polyethylenimine

**DOI:** 10.1155/2011/204058

**Published:** 2011-03-21

**Authors:** Jennifer A. Fortune, Tatiana I. Novobrantseva, Alexander M. Klibanov

**Affiliations:** ^1^Department of Chemistry, Massachusetts Institute of Technology, Cambridge, MA 02139, USA; ^2^Alnylam Pharmaceuticals, Cambridge, MA 02139, USA; ^3^Department of Biological Engineering, Massachusetts Institute of Technology, Cambridge, MA 02139, USA

## Abstract

We mechanistically explored the effect of increased hydrophobicity of the polycation on the efficacy and specificity of gene delivery in mice. *N*-Alkylated linear PEIs with varying alkyl chain lengths and extent of substitution were synthesized and characterized by biophysical methods. Their *in vivo* transfection efficiency, specificity, and biodistribution were investigated. *N*-Ethylation improves the *in vivo* efficacy of gene expression in the mouse lung 26-fold relative to the parent polycation and more than quadruples the ratio of expression in the lung to that in all other organs. *N*-Propyl-PEI was the best performer in the liver and heart (581- and 3.5-fold enhancements, resp.) while *N*-octyl-PEI improved expression in the kidneys over the parent polymer 221-fold. As these enhancements in gene expression occur without changing the plasmid biodistribution, alkylation does not alter the cellular uptake but rather enhances transfection subsequent to cellular uptake.

## 1. Introduction

The promise of gene therapy has yet to be realized for lack of a safe, efficacious, and specific delivery vector [1]. Viral vectors are naturally equipped to evoke maximal gene expression but have failed to prove safety in clinical trials [1–3]. In contrast, cationic polymers and lipids demonstrate superior safety but do not afford clinically relevant levels of gene expression [4–7]. Polyethylenimine (PEI) is considered a leading polycationic vector for gene delivery [8]. Work in our lab and others has demonstrated successful *in vivo* nucleic acid delivery in mice with various substituted PEIs [8–10]. 

Branched PEI first emerged as a “gold standard” of nonviral gene delivery in terms of efficacy but was plagued by toxicity concerns [11]. Less toxic lower molecular weight branched PEIs, specifically those of 2-kDa molecular weight, failed to afford sufficient gene expression [12–15]. However, *N*-dodecylation of this PEI dramatically enhanced the protein expression mediated by it to levels at least comparable to those of the 25-kDa polycation [16]. More recently, linear 22-kDa PEI has emerged as a premier PEI for gene delivery [10, 17]: it exhibits a 21-fold enhancement in protein expression over leading branched PEIs but with minimal toxicity [10].

In the present study, we synthesized and mechanistically explored *N*-alkylated linear PEIs for their ability to enhance and alter the specificity of gene delivery *in vivo* in mice. We found that covalent derivatization of a small fraction of PEI's amino groups with short-chained alkyls enables a 26-fold enhancement of gene expression in the mouse lung while increasing the amount of expression in this organ relative to others some 4-fold. Interestingly, the effect of alkylation varied among tissues and did not alter uptake of polyplexes into cells; rather, it seemed to affect an intracellular transfection step.

## 2. Materials and Methods

### 2.1. Chemicals and NMR

Na^125^I was purchased from Perkin Elmer. All other chemicals were from Sigma-Aldrich (St. Louis, MO) and were of the highest purity available. NMR spectra were recorded using a Bruker 400-MHz NMR spectrometer with chemical shifts expressed with reference to the chloroform peak in CDCl_3_ (7.24 ppm).

### 2.2. Plasmid and Its Lodination

gWiz Luc encoding the firefly luciferase gene was purchased from Aldevron (Fargo, ND). This ready-to-use plasmid, containing the luciferase gene under the control of a modified promoter from the cytomegalovirus immediate early gene, was obtained as a 5.0 mg/mL stock solution in water.

Iodination of the plasmid was completed using a modified version of previously described methods [18, 19]. Briefly, two iodobeads from Thermo Scientific (Rockford, IL) were incubated with 30 *μ*L of 0.35 M sodium acetate buffer, pH 4.0, and 400 pmol of Na^125^I (containing 1 mCi of radiation) at 50°C for 15 min. To that, 100 *μ*g of gWiz Luc (in 20 *μ*L) was added and incubated for an additional 30 min. The iodinated plasmid was purified by sequential desalting on Minitrap and Miditrap desalting columns (GE Healthcare).

### 2.3. Linear PEI Synthesis and Its N-Alkylation

Fully deacylated linear PEI of 22-kDa was synthesized from commercial 200-kDa poly(2-ethyl-2-oxazoline), PEOZ, as previously described [10]. Briefly, 3.0 g of the PEOZ was added to 120 mL of 24% (w/v) HCl followed by refluxing for 96 h. The PEOZ crystals dissolved completely in 2 h, but 3 h later a white precipitate appeared. The solution was brought to pH 10 with 10 M NaOH, isolated by vacuum filtration, washed with cold water, and lyophilized as the free base. The resultant white powder was confirmed by NMR to be pure PEI base.

The linear PEI obtained was reacted with 10 mol% of iodomethane, iodoethane, iodopropane, iodobutane, or iodooctane in ethanol as previously described [16]. The *N*-alkylated crude products, obtained upon rotary evaporation, were dissolved in water, adjusted to pH 10 with 10 M NaOH, isolated by vacuum filtration, washed with cold water, and lyophilized. Pure products (~11% alkyl group substitution) were obtained as white hygroscopic solids on lyophilization. ^1^H NMR spectroscopy (with CDCl_3_ as an internal standard) was used to determine the percent of *N*-alkylation through comparison of the integration of the 0.5–1 ppm triplet from the protons of the terminal methyl group of the alkane with the 2.5–3 ppm peak of the polymer's ethylene protons.

### 2.4. pH Titration

Acid titrations were carried out using a 1-mL solution of each *N*-alkyl-PEI (113 mM in –CH_2_CH_2_NH– units) adjusted to pH 11.5 with NaOH. Sequential additions of 20-*μ*L volumes of 0.5 M HCl were performed, and the pH after each addition was measured; 113 mM aqueous NaCl was titrated similarly as a control using 0.04 M HCl. All experiments were conducted in triplicate.

### 2.5. Ethidium Bromide (EtBr) Displacement Assay

To a 200 *μ*L solution of gWiz Luc DNA (4.1 *μ*g) and EtBr (0.63 *μ*g) in 10 mM PBS in a black 96-well plate, 5-*μ*L aliquots of 0.63 mM *N*-alkyl-PEI stock solutions were added sequentially. Fluorescence spectra of free EtBr (*F*_*I*_), of EtBr/DNA (*F*_*D*_), and of EtBr/DNA after each addition of PEI (*F*_*C*_) were recorded (*λ*_ex_ = 523 nm and *λ*_em_ = 587 nm). Relative fluorescence values were calculated using the formula [(*F*_*C*_ − *F*_*I*_)/(*F*_*D*_ − *F*_*I*_)] × 100%.

### 2.6. Gene Delivery in Mice

All animal experiments conducted in this study adhered to the Principles of Laboratory Animal Care (National Institutes of Health publication no. 85-23, revised in 1985) and were approved by the Institutional Committee on Animal Care. To obtain an *N*/*P* ratio of 8 (that of PEI nitrogen to DNA phosphate), appropriate volumes of *N*-alkyl-PEI stock solutions were diluted to 500 *μ*L in 5% aqueous glucose and added to an equal volume of the glucose solutions containing 350 *μ*g of the plasmid DNA (gWiz Luc) followed by pipette mixing. An *N*/*P* ratio of 8 was selected because it was previously determined to be optimal for linear PEI delivery *in vivo* in mice. The resulting polyplexes were incubated at room temperature for 10 min. Then 6- to 8-week-old Swiss Webster female mice (Taconic Farms) were injected intravascularly via tail vein with 200 *μ*L of the polyplexes containing 70 *μ*g of DNA. After 24 h, the mice were euthanized by CO_2_ inhalation; their lungs, kidneys, livers, hearts, and spleens were collected, washed with PBS, and suspended in lysis buffer prepared by mixing 4 mL of 5x passive lysis buffer (Promega), 800 *μ*L of 8.7 mg/mL phenylmethylsulfonyl fluoride (PMSF) in methanol, 400 *μ*L of protease inhibitor mixture, and 14.8 mL of water. The samples were freeze-thawed, homogenized by probe-sonication for 40 sec in 20-second intervals, and centrifuged. Then 10 *μ*L of the supernatants was mixed with 100 *μ*L of the luciferase assay reagent (Promega), and the luminescence was measured using an Optocomp I luminometer (MGM Instruments, Hamden, CT). Protein concentrations were determined using the bicinchoninic acid (BCA) assay, and the results were expressed as mean ± SD (*n* = 4).

### 2.7. Mouse Perfusion and Radiation Measurements

Polyplexes were prepared as described in the previous section. At 5, 10, 15 min, or 24 h, mice were anesthetized with a lethal dose of Avertin, the vena cava was cut, a blood sample was collected, and the animals were perfused with PBS using a peristaltic pump at a flow rate of about 20 mL/min for 5–10 min. Once the perfusion ran clear, the organs were dissected and assayed for gamma counts using 5-minutes read time. The results were expressed as mean ± SD (*n* = 4).

## 3. Results and Discussion

While much has been done to improve its transfection efficiency, linear PEI, currently a “gold standard” of polycationic gene delivery, still falls short of characteristics required for clinical utility. In this work, we prepared a series of *N*-alkylated linear PEI derivatives with the goal of developing a more efficient and specific vector for *in vivo* transfection. In particular, methyl-PEI, ethyl-PEI, propyl-PEI, butyl-PEI, and octyl-PEI were synthesized from the corresponding iodoalkanes and fully deacylated linear PEI. Alkylation conditions were optimized to derivatize approximately 11% of the backbone amines for all derivatives (as determined by H^1^ NMR spectroscopy). This level of modification was experimentally determined to be the highest (and most efficacious) degree of substitution that did not result in precipitation of the polyplexes.

**Figure 1:**
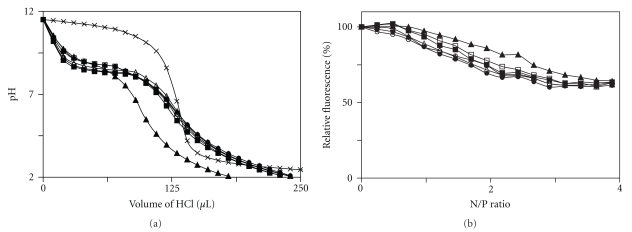
The effect of *N*-alkylation on the buffer capacity and DNA binding efficiency of linear PEI. (a) Acid titration profiles of aqueous solutions of the underivatized PEI (solid squares), methyl-PEI (open squares), ethyl-PEI (solid triangles), propyl-PEI (open triangles), butyl-PEI (solid circles), octyl-PEI (open circles), and NaCl as a control (× symbols). The corresponding 113 mM solutions were adjusted to pH 11.5 at room temperature and then titrated with 0.5 M HCl (0.04 M in the case of NaCl). (b) Displacement of the intercalated fluorophore EtBr from plasmid DNA by the underivatized PEI (solid squares), methyl-PEI (open squares), ethyl-PEI (solid triangles), propyl-PEI (open triangles), butyl-PEI (solid circles), and octyl-PEI (open circles). *N*/*P* ratio is that between the nitrogen atoms in the polycation and the phosphate groups of the bases in the plasmid.

The ability to buffer endosomes/lysosomes and condense DNA is a necessary requirement for efficient gene delivery by polycationic vectors. To assess the effect of *N*-alkylation on the ability of PEI to mediate these steps, the PEIs were characterized by acid titration and ethidium bromide (EtBr) displacement from plasmid DNA. As seen in Figure 1(a), all *N*-alkylated PEIs retain significant buffering capacity. Likewise, the derivatives all condense plasmid DNA to an appreciable degree (Figure 1(b)). Under the conditions used, polyplexes are stable for at least 1 h. 

It is interesting to note that *N*-ethyl-PEI demonstrates reduced DNA condensation and buffering capacity, suggesting that the fluid phase dynamics and DNA interactions of alkylated polyamines is complicated and further research is required to fully understand the relationship between the chemical structure of *N*-alkylated linear PEIs and their biophysical properties. This sentiment has been mirrored for other polyionic gene delivery systems in recent years [20–22].

Both condensation/decondensation of DNA and endosomal/lysosomal buffering are critical steps in cell transfection [7, 23]; while ideal conditions for these steps have not been established, it is known that effective DNA condensation to form polyplexes and subsequent decondensation inside the cell are in direct competition [24]. Therefore, these data alone are not sufficient to predict the transfection properties of ethyl-PEI or any of the other *N*-alkylated PEI derivatives [25].

**Figure 2:**
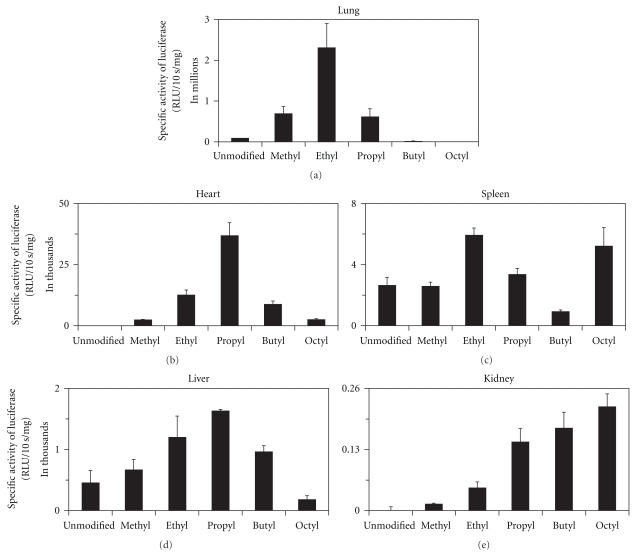
Comparison of the gene expression in the lungs (a), heart (b), spleen (c), liver (d), and kidneys (e) of a plasmid containing the luciferase gene mediated by the following *N*-alkylated linear PEI derivatives: (i) unmodified, (ii) methyl, (iii) ethyl, (iv) propyl, (v) butyl, and (vi) octyl.

Since our unpublished work pointed to a lack of correlation between in vitro (i.e., in cell culture) and *in vivo* (i.e., in animal models) PEI-mediated gene expression, the *N*-alkylated linear PEI derivatives were investigated for their ability to efficiently and specifically transfect cells only *in vivo*. Figure 2 depicts the gene expression profiles in mice of the parent linear PEI and its *N*-alkylated derivatives. One can see that in the lung, the tissue which demonstrates over 96% of all luciferase expression for the parent polycation, methyl-PEI, ethyl-PEI, and propyl-PEI exhibited enhanced luciferase expression relative to the parent by 8-, 26-, and 7-fold, respectively (Figure 2(a)). In contrast, longer alkyl chains negatively affected the transfection efficiency: while pulmonary luciferase expression mediated by butyl-PEI is marginally reduced, octyl-PEI demonstrates 200-fold lower expression in the lung than the unmodified PEI (Figure 2(a)). 

Interestingly, the other mouse tissues examined each demonstrate a unique expression profile with respect to the alkyl chain length (Figure 2). In the heart and liver, propyl-PEI performs the best by 3.5- and 581-fold, respectively, over the parent polycation (Figures 2(b) and 2(d)) while in the kidneys longer alkyl chain mediates enhanced gene expression with octyl-PEI providing 221-fold greater expression than unmodified PEI (Figure 2(e)). In the spleen, ethyl- PEI and octyl- PEI produce similar levels of luciferase expression that are twice those of the parent's (Figure 2(c)). It is unclear why this unique trend is observed in the spleen, warranting further investigation.

The changes in the luciferase expression profile can be summarized by the ratio of expression in the lungs relative to all other organs combined. While for the parent PEI that ratio is 28 : 1, it jumps to 119 : 1 and 117 : 1, respectively, for the methyl and ethyl derivatives. For the three remaining derivatives (propyl- PEI, butyl- PEI, and octyl-PEI), however, the ratio drops to 15 : 1, 2 : 1, and 0.06 : 1, respectively. In the octyl-PEI-mediated delivery, where lung expression does not dominate, maximal expression is seen in the spleen. This suggests that *N*-alkylation can be used to modulate the organ specificity of gene expression. 

**Figure 3:**
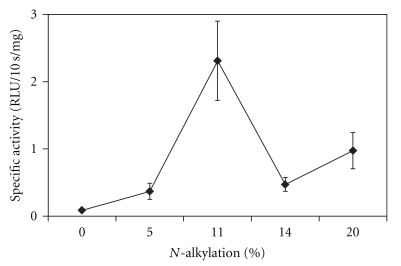
Gene expression in the lungs of a plasmid containing the luciferase gene mediated by *N*-ethyl-PEI as a function of the degree of the polycation's alkylation.

Given the dramatic differences in protein expressions across mouse organs with 11% alkylation, we investigated the effect of varying degrees of *N*-alkylation with the best performing ethyl-PEI. As seen in Figure 3, the 11% alkylation happens to produce the most efficient gene expression in the lungs as compared with the 5%, 14%, and 20% derivatization. Higher degrees of *N*-alkylation of linear PEI were not tested due to drastically diminished solubility of the resultant polycations.

Since protein expression is ultimately required for gene therapy to become a clinical reality, it is an appropriate endpoint measurement. However, it is also of mechanistic interest to establish in what organ the delivered plasmid ends up. To this end, we treated mice with ^125^I-labeled DNA in complexes with the *N*-alkylated linear PEI derivatives to follow the localization of the delivered plasmid. The biodistribution of the polyplexes was determined *in vivo* at 24 h, the time point of the efficiency study, as shown in Table 1. At 24 h, most of the plasmid had been excreted; what remained resided predominantly in the clearance organs, specifically the kidneys and liver (0.1–0.5% of injected dose for the best transfecting ethyl-PEI). Less than 0.01% of the injected dose remained in the lung at 24 h regardless of the delivery vector.

**Figure 4:**
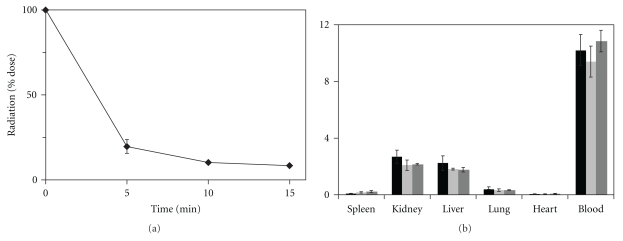
(a) Pharmacokinetic profile of ^125^I-labeled gWiz Luc plasmid DNA with no delivery vector with an initial dose of 70 *μ*g of the plasmid containing approximately 1 *μ*Ci of radiation. (b) Comparison of the organ biodistribution of ^125^I-labeled plasmid containing the luciferase gene complexed with no vector (black), linear PEI (light grey), and *N*-ethylated linear PEI (dark grey) at 15 minutes.

Elucidating the biodistribution of the plasmid at an earlier time point may be critical to understanding the effect of *N*-alkylation of linear PEI on localization of the delivered plasmid. Since the biodistribution of naked plasmid and PEI-mediated plasmid delivery were identical, the pharmacokinetics of naked plasmid clearance was evaluated to determine the optimal time point for this analysis. As seen in Figure 4(a), nearly 80% of the free plasmid was cleared from circulation in just 5 min and some 90% at 15 min. Given the rapid clearance from the blood, the biodistribution was subsequently assessed at 15 min. 

As seen in Figure 4(b), the tissue distribution of naked plasmid DNA, linear PEI, and the most competent *N*-alkylated derivative (ethyl-PEI) at 15 min is very similar to that at 24 h. The kidneys and liver retained the greatest percentages of delivered radiation (about 2% of the injected dose each for ethyl-PEI) while the lung retained much less (only 0.35% of the injected dose). Interestingly, delivery of plasmid via a polycationic vector does not change the biodistribution of the plasmid at either 15 min or 24 h as compared to naked plasmid. Moreover, even clearance from the blood occurs at the same rate. 

We previously hypothesized that enhanced interactions with cell membranes due to increased hydrophobicity stemming from *N*-alkylation of branched PEI could enhance polyplex uptake and subsequent gene expression [16]. However, studies described herein revealed no appreciable change in the biodistribution of free plasmid DNA relative to that delivered with a vector, either linear PEI itself or its *N*-ethylated derivative. The localization of ^125^I-labeled plasmid DNA is similar across all tissues and all polycationic vectors at 24 h and across all those investigated at 15 min (Figure 4(b)). This suggests that at least in the case of linear PEI, its *N*-alkylation does not enhance uptake of the polyplexes and instead plays some other, yet to be determined role. 

Note that expression of protein from delivered plasmid DNA requires the successful completion of several steps: (i) uptake by endocytosis; (ii) endosomal/lysosomal escape; (iii) transport to, and uptake by, the nucleus; (iv) decondensation of DNA from the polycation; (v) transcription and translation of delivered plasmid. Given the changes to buffering capacity and DNA condensation seen with *N*-ethylated PEI and the greatly enhanced protein expression observed as a result, it is likely that either the second or the fourth of these steps is enhanced by ethylation.

## 4. Conclusions

In closing, herein we have examined *in vivo* transfection efficiency, specificity, and biodistribution of a model plasmid mediated by *N*-alkylated linear PEIs with varying alkyl chain lengths and extent of substitution. While *N*-ethylation greatly improves the efficacy of gene expression in the mouse lung relative to that by the parent polycation, *N*-propyl-PEI and *N*-octyl-PEI are dramatically better performers than PEI in the liver and kidneys, respectively. Since these enhancements in gene expression occur without changing the plasmid biodistribution, we conclude that alkylation of PEI enhances transfection subsequent to cellular uptake rather than the latter process itself.

## Figures and Tables

**Table 1:** Biodistribution of ^125^I-labeled gWiz Luc plasmid delivered with linear PEI and its *N*-alkylated derivatives. Values are the percentages of injected dose still remaining in select tissues at 24 h.

Polycation	Spleen	Kidney	Liver	Lung	Heart	Blood
unmodified PEI	0.03	0.11	0.49	0.01	0.00	0.02
*N*-methyl-PEI	0.02	0.08	0.30	0.01	0.00	0.03
*N*-ethyl-PEI	0.02	0.12	0.30	0.02	0.01	0.06
*N*-propyl-PEI	0.02	0.11	0.30	0.01	0.00	0.03
*N*-butyl-PEI	0.02	0.09	0.29	0.01	0.00	0.03
*N*-octyl-PEI	0.03	0.05	0.27	0.01	0.00	0.01

## Abbreviations

PEI: Polyethylenimine
EtBr: Ethidium bromide.
